# The Discovery of Membrane Vesicle Biogenesis in the Polyhydroxybutyrate-non-producing Mutant Strain of *Cupriavidus necator* H16

**DOI:** 10.1264/jsme2.ME24007

**Published:** 2024-09-25

**Authors:** Sangho Koh, Michio Sato, Hiromi Matsusaki, Seiichi Taguchi

**Affiliations:** 1 Graduate School of Science, Technology and Innovation, Kobe University, 1–1 Rokkodai-cho, Nada-ku, Kobe, Hyogo 657–8501, Japan; 2 School of Agriculture, Meiji University, 1–1–1 Higashimita, Tama, Kawasaki, 214–8571, Japan; 3 Faculty of Environmental and Symbiotic Science, Prefectural University of Kumamoto, 3–1–100 Tsukide, Higashi-ku, Kumamoto 862–8502, Japan; 4 Engineering Biology Research Center, Kobe University, Nada, Kobe, Hyogo 657–8501, Japan

**Keywords:** *Ralstonia eutropha*, envelope stress, blebbing, polyhydroxyalkanoate, bioplastic

## Abstract

Extracellular membrane vesicles (MVs) caused by the artificial production of polyhydroxybutyrate (PHB) were previously detected in *Escherichia coli*. We herein observed MV biogenesis in the mutant strain (–PHB) of the natural PHB producer, *Cupriavidus necator* H16. This inverse relationship was revealed through comparative electron microscopic ana­lyses of wild-type and mutant strains. Based on these results, we speculate that a physiological relationship exists between MV biogenesis and PHB biosynthesis. Therefore, we propose the potential of MV biogenesis as a fermentative “stress marker” for monitoring the performance of target polymer-producing microbial platforms.

Membrane vesicles (MVs) are lipid nanoparticles with diameters that range between 20 and 400‍ ‍nm and are released from the cell surfaces of various microorganisms. Microbial MVs serve as versatile cargoes with multifunctional properties, including horizontal gene transfer, the export of cellular metabolites and virulence, phage infection, and cell-to-cell communication (Toyofuku *et al.*, 2023). Recent studies reported the use of MVs in the development of drug delivery carriers and applications in nanotechnology. MVs are generated in Gram-negative bacteria through cell membrane budding triggered by “envelope stress”, such as lipid accumulation (Roier *et al.*, 2016), peptidoglycan damage (McBroom *et al.*, 2006), and misfolded protein accumulation in the periplasmic space (McBroom and Kuehn, 2007).

We were the first to report the biogenesis of MVs in *Escherichia coli* due to the recombinant production of the biodegradable polyester, polyhydroxybutyrate (PHB), the most extensively exami­ned polyhydroxyalkanoate (PHA) (Koh *et al.*, 2022). The PHB biosynthetic pathway was constructed in *E. coli* by installing operon genes encoding β-ketothiolase (PhaA), NADPH-dependent reductase (PhaB), and PHB synthase (PhaC) derived from *Cupriavidus necator* H16 (Fig. 1A). The intracellular accumulation of PHB was considered to be a trigger for MV biogenesis in this artificial microbial system. The secreted level of MVs was proportional to the accumulated level of PHB in recombinant cells. The relationship between MV formation and PHB production can be regulated by varying the concentration of glucose, suggesting a causal relationship between these useful biomaterials artificially produced in‍ ‍the microbial platform. We subsequently designated this‍ ‍new phenomenon as the **P**olymer **I**ntracellular **A**ccumulation-triggered system for **MV** **P**roduction, also referred to as “PIA-MVP” (Koh *et al.*, 2022). PIA-MVP is a promising microbial platform that allows us to conduct further studies with a focus on biopolymer encapsulation and cross-membrane transportation for different applications. We demonstrated the efficient encapsulation of the cytoplasmic protein, green fluorescent protein (GFP), in the form of multi-layered MVs involving the inner membrane, as revealed by transmission electron microscopy (TEM) (Koh *et al.*, 2023). Using the established GFP-monitoring system, we easily created a regulated condition to selectively produce single-layered MVs and a mixture of single- and multi-layered MVs by controlling the concentration of glucose (Koh *et al.*, 2023).

In the present study, we focused on MV biogenesis in the natural PHB producer, *C. necator* H16. *C. necator* (previously known as *Wautersia eutropha*, *Ralstonia eutropha*, and *Alcaligenes eutrophus*) is a Gram-negative bacterium belonging to the *Burkholderiaceae* family. *C. necator* cells grow and accumulate PHB by utilizing organic carbon sources, such as fructose, glycolate, citrate, and acetate, as well as inorganic carbon sources, including CO_2_ (Panich *et al.*, 2021). Strain H16 is a robust platform for the industrial production of the evolved version of PHB, P(3HB-*co*-3-hydroxyhexanoate) (PHBH) manufactured by Kaneka. We herein unexpectedly detected MV biogenesis during the cultivation of the PHB-non-producing mutant strain (–PHB) of *C. necator* H16, as previously observed for cultivated cells of the PHB-producing strain of *E. coli* (Koh *et al.*, 2022). This microscopic observation was the starting point our investigation of MV biogenesis in *C. necator* H16. The experimental procedures performed are described as follows.

The wild-type and –PHB strains were cultivated in 500-‍mL shaking flasks containing 100‍ ‍mL modified basal mineral medium supplemented with 20‍ ‍g‍ ‍L^–1^ fructose as the sole carbon source, and were incubated at 30°C for 3 days. The modified basal mineral medium comprised 1.10% w/v Na_2_PO_4_·12H_2_O, 0.19% w/v KH_2_PO_4_, 0.129% w/v (NH_4_)_2_SO_4_, 0.1% w/v MgSO_4_·7H_2_O, and 0.1% v/v trace element solution (0.1 M HCl solution with 1.6% w/v FeCl_2_·6H_2_O, 1% w/v CaCl_2_·2H_2_O, 0.02% w/v CoCl_2_·6H_2_O, 0.016% w/v CuSO_4_·5H_2_O, and 0.012% w/v NiCl_2_·6H_2_O). The PHB synthetic gene operons encoding PhaC1 (H16_A1437), PhaA (H16_1438), and PhaB (H16_1439) were eliminated via homologous recombination as described in a previous study (Mifune *et al.*, 2008). We confirmed the intracellular accumulation of PHB granules in the wild-type strain using the Nile red staining method, as shown in a fluorescence microscopic image (Fig. 1B, left side). On the other hand, as expected upon the deletion of the PHB synthetic gene operons, the mutant strain did not accumulate PHB granules (Fig. 1B, right side). Interestingly, a critical difference was observed between the wild-type and non-PHB-producing strains. Budded particles occurred specifically on the cell membrane surface of the PHB-non-producing mutant (Fig. 2A, right side), but not for the wild-type strain. This unique surface morphological change was consistent with our previous findings on the PHB-producing strain of *E. coli* (Koh *et al.*, 2022). Based on this phenomenon, we assumed the presence of extracellular MVs. Therefore, we attempted to address this proof-of-concept of MV biogenesis by investigating extracellular fractions.

To confirm extracellular MVs, bacterial cells were removed from culture broths by centrifugation at 6,000×*g* for 5‍ ‍min followed by filtration through the membrane filter ADVANTEC DISMIC-25CS^®^ (Tokyo Roshi) with a pore size of 0.45‍ ‍μm, according to a previously reported method (Koh *et al.*, 2022). The experimental sample was collected from 20‍ ‍mL of filtered culture supernatants by ultracentrifugation in a polycarbonate bottle with a cap assembly (25×89‍ ‍mm, 26.3‍ ‍mL; Beckman Coulter) at 100,000×*g* at 4°C for 60‍ ‍min in Optima^TM^ LE-80K with a Type 70Ti rotor (Beckman Coulter). Further sample purification was performed by sequential size exclusion column chromatography using a qEV 35‍ ‍nm Gen2 column (Izon), according to a previous study (Hong *et al.*, 2019). The column was washed with 30‍ ‍mL of phosphate-buffered saline (PBS) and a crude MV sample (0.5‍ ‍mL) was applied to the column. Twelve milliliters of PBS was eluted into the column, and 0.5-mL fractions were collected manually. Eluted fractions were stored at –30°C. The collected sample was dissolved in 100‍ ‍μL of PBS and incubated with 1.25‍ ‍μg mL^–1^ of the fluorescent lipid probe FM4-64 (Invitrogen) in PBS. Fluorescence intensity was measured at excitation and emission wavelengths of 558 and 734‍ ‍nm, respectively, using a microplate fluorescence reader (Tecan Group).

The formation of nano-sized vesicles was clearly observed for the first time in the TEM image shown in Fig. 2B. The average diameter of the vesicles was subsequently measured by dynamic light scattering using Zetasizer Ultra (Malvern Panalytical; Fig. 2C). The average particle diameter of MVs from the –PHB strain was 118.5‍ ‍nm and the average concentration of MVs was estimated to be 2.10×10^9^ particles mL ^–1^, which were consistent with the values obtained for PHB-producing *E. coli* in our previous study (Koh *et al.*, 2022). In contrast, no MV particles were detected in the PHB-producing H16 strain. These vesicles were identified as lipid-based particles based on the fluorescence intensity observed using the fluorescent lipid probe FM4-64. These results suggest that the –PHB strain secreted MVs extracellularly.

To confirm whether MV generation occurred due to an inability to produce PHB, a complementation ana­lysis was performed (Fig. 3). A complementary strain (–PHB::pBBR-CAB_Cn_), in which the ability to produce PHB was restored, was constructed by introducing the plasmid pBBR-CAB_Cn_, which carried the PHB synthetic gene operons phaC1, phaA, and phaB (Miyahara *et al.*, 2022), into the –PHB strain (Fig. 3A). Consistent with the wild-type H16 strain, MV generation was not detected in the complementary strain –PHB::pBBR-CAB_Cn_, indicating that MV biogenesis occurred due to an inability to produce PHB (Fig. 3B). Based on these microscopic and biochemical ana­lyses, we concluded that this is the first study to show MV biogenesis in the natural producer of PHB, *C. necator* H16. In contrast to the –PHB mutant, fluorescence was not detected in the extracellular fraction of the H16 strain or –PHB::pBBR-CAB_Cn_, the strain that regained the ability to produce PHB.

The mode of MV biogenesis is highly diverse and is dependent on the physiological state of the corresponding microbes (Toyofuku *et al.*, 2023). To date, a substantial body of literature has been accumulated from the perspective of natural ecosystems, such as biofilm formation (Turnbull *et al.*, 2016), and medical applications, including vaccine development (Lieberman, 2022). Microbe-based synthetic biology aimed at producing value-added products has recently been extensively exami­ned in consideration of cell growth and target production. In this biotechnological approach, we often encounter the development of genetic circuits, including toggle switches, to overcome the carbon-flux-utilization competition issue between cell growth and target production (Gardner *et al.*, 2000; Soma *et al.*, 2014). Therefore, the cellular physiology of microbial platforms needs to be managed in order to optimize production systems. We propose the potential of MV biogenesis as a fermentative stress marker for monitoring the performance of target-producing microbial platforms.

In the long stream of PHA research, the occurrence of MVs due to the elimination of PHB production was herein observed for the first time for the industrial biopolymer producer, *C. necator* H16. A related study on the physiological relationship between PHB production and the appearance of blebs referred to the alginate secretory producer *Azotobacter vinelandii* (Hashimoto *et al.*, 2013; Yoneyama *et al.*, 2015). Although the terminology for MVs was not clearly described in these studies, our observation experiences indicate that a few blebs on the cell surface in a scanning electron microscope image may be MVs. A positive relationship was observed between PHB production and the formation of MV-like particles, which is similar to PIA-MVP in the *E. coli*-based artificial system (Koh *et al.*, 2022). This common event provides insights into the mechanisms underlying PIA-MVP. Interestingly, we herein found an inverse relationship between both supramolecules, PHB and MVs, in *C. necator* H16, as shown in Fig. 4. PHA is generally considered to be a carbon storage material. Moreover, additional functions as physiological protectants against external stresses, such as osmotic and thermal shocks, have been reported (Obruca *et al.*, 2016; Sedlacek *et al.*, 2019; Müller-Santos *et al.*, 2021). The occurrence of MVs has been attributed to envelope stress caused by external and/or internal physiological changes (Tashiro, 2022). In brief, if MV biogenesis is a stress signal for living microorganisms, PHB synthesis may be in a normal state for *C. necator*, but not for *E. coli*. It is important to note that the presence of granule-coating proteins (phasins) forming a layer at the intracellular PHB granule surface may prevent a physiologically stressful attachment between cellular components and PHB granules in the natural PHB-producing *C. necator* H16 (Mezzina and Pettinari, 2016). Therefore, the presence/absence of phasins may be one of the reasons for the physiological discrepancy in the mode of MV biogenesis between *C. necator* H16 and *E. coli*. In further studies, the mole­cular mechanisms underlying PHB synthesis-associated MV biogenesis need to be clarified based on this hypothesis.

The significance of PHB production in *C. necator* H16 may be discussed based on its relationship with several physiological stressors by monitoring MV biogenesis as a stress marker. Robustness in the fermentation process is crucial for the industrial production of value-added products, such as bioplastics, using microbial platforms, including *C. necator* H16. *C. necator* H16 is a powerful platform for the production of natural PHB and the unnatural PHBH copolymer, named GreenPlanet^TM^, which is commercially manufactured at 20,000 tons year^–1^ by Kaneka. Most recently, we have been upgrading the *C. necator* H16-based platform for the overproduction of P(lactate-*co*-3HB), termed LAHB (Taguchi *et al.*, 2008; Imai *et al.*, 2024; Koh *et al.*, 2024), as an evolved version of polylactide, which is commercially used in various applications as a representative bio-based polyester. The present results on the PHB-MV relationship need to considered to achieve further advances in this biopolymer industrialization project.

In conclusion, this is the first study to demonstrate the secretion of MVs from the mutant strain of chemotrophic *C. necator* H16 strain that originally produced the biodegradable polyester PHB from renewable feedstocks. We propose the potential of MV biogenesis as a fermentative stress marker for monitoring the performance of target polymer-producing microbial platforms. This discovery has prompted us to conduct an in-depth investigation of the relationship between MV biogenesis and PHB biosynthesis in *C. necator* H16 from the viewpoints of basic research and industrial applications.

## Citation

Koh, S., Sato, M., Matsusaki, H., and Taguchi, S. (2024) The Discovery of Membrane Vesicle Biogenesis in the Polyhydroxybutyrate-non-producing Mutant Strain of *Cupriavidus necator* H16. *Microbes Environ ***39**: ME24007.

https://doi.org/10.1264/jsme2.ME24007

## Figures and Tables

**Fig. 1.**
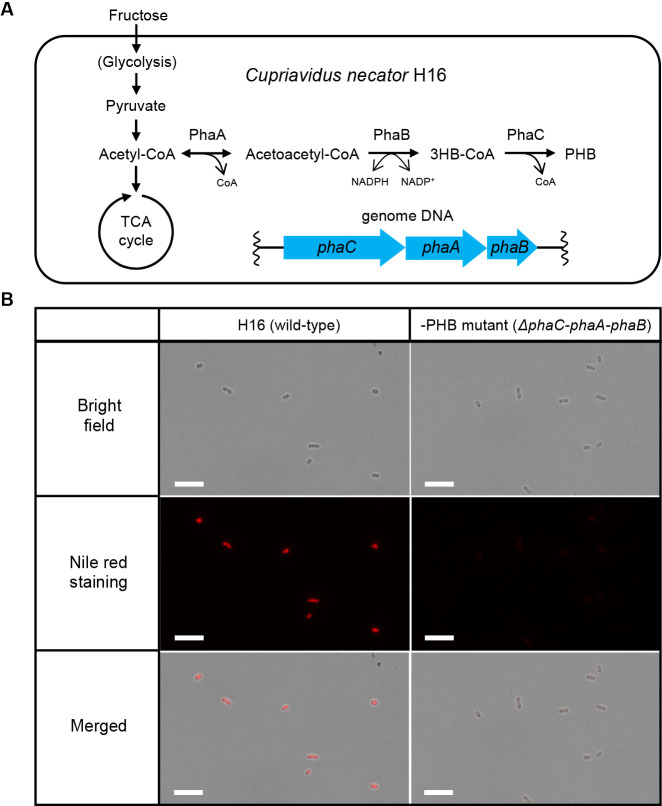
(A) Metabolic pathway for the biosynthesis of PHB in *Cupriavidus necator* H16. Acetyl-CoA supplied from fructose via glycolysis is converted to acetoacetyl-CoA through the function of β-ketothiolase (PhaA). Acetoacetyl-CoA is converted into 3HB-CoA by the function of NADPH-dependent-CoA reductase (PhaB). The resultant 3HB-CoA molecules are polymerized to PHB by the function of PHA-synthase (PhaC). (B) Fluorescent microscopic images of the wild-type and –PHB mutant strains. When operon genes encoding PhaC, PhaA, and PhaB were disrupted (named the –PHB strain), the production of PHB was not observed. Scale bars represent 5‍ ‍μm.

**Fig. 2.**
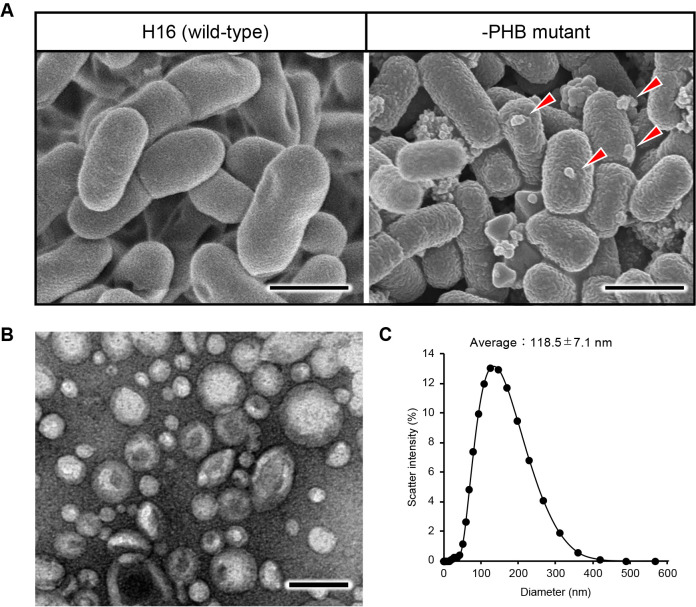
(A) Budding from the cell surface of the PHB non-producing mutant strain (–PHB) revealed by scanning electron microscopy (SEM). Red arrowheads indicate budding from the cell surface. Scale bars represent 1‍ ‍μm. (B) Transmission electron microscopy (TEM) images and (C) the size distribution of purified MVs isolated from the culture supernatant of the –PHB mutant strain. Scale bars represent 100‍ ‍nm.

**Fig. 3.**
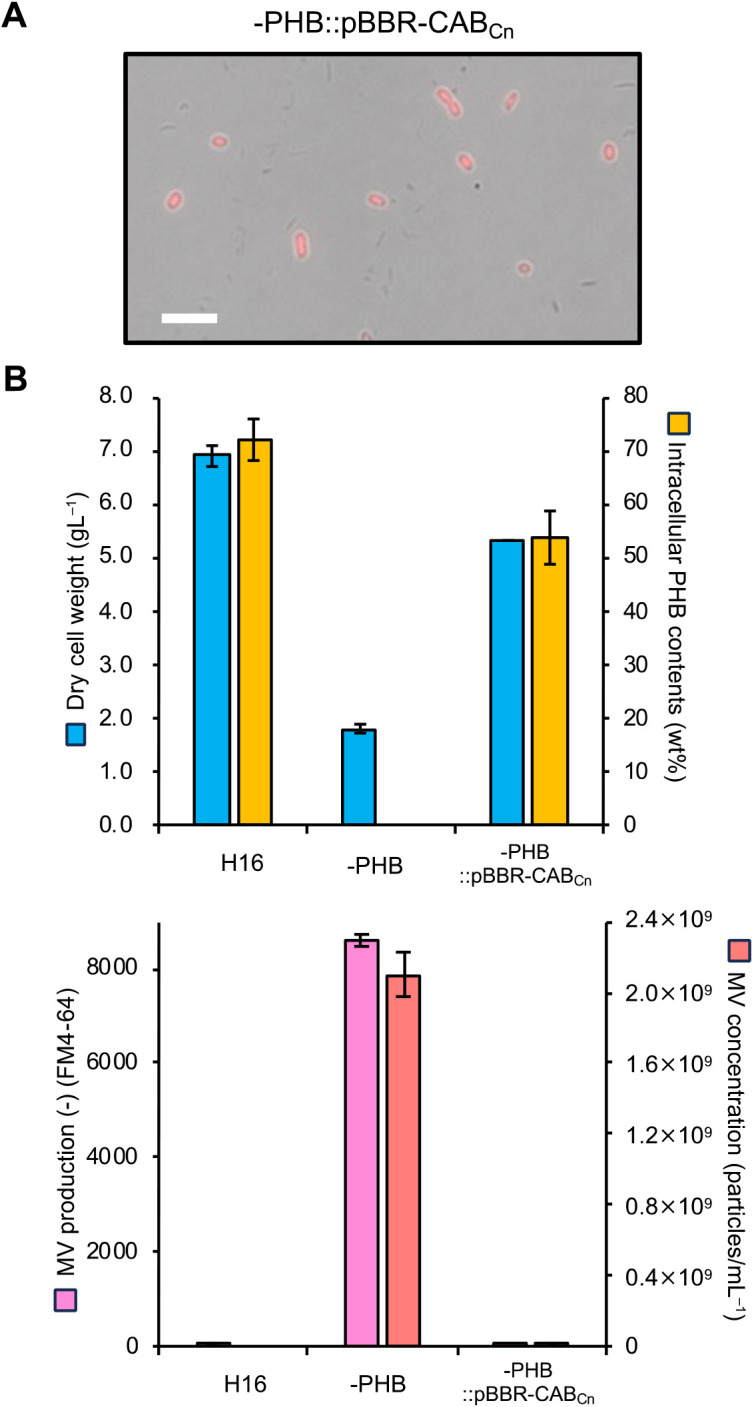
Complementary ana­lysis of the –PHB strain. (A) Confirmation of the regained ability of the complementary strain harboring pBBR-CAB_Cn_ (–PHB:: pBBR-CAB_Cn_) to produce PHB by fluorescent microscopy. Scale bars represent 5‍ ‍μm. (B) Relationship between intracellular PHB production and extracellular MV production in the following three strains: H16, –PHB mutant, and –PHB:: pBBR-CAB_Cn_. After cultivation at 30°C for 72 h, collected cells were freeze-dried and intracellular PHB contents were measured by gas chromatography, as previously described (Koh *et al.*, 2022). Extracellular MVs were collected from the culture supernatant and quantified by fluorescent intensity with the lipid probe FM4-64 and dynamic light scattering measurements. Data are presented as the means of triplicate experiments, and error bars represent standard deviations. Data are presented as the means of triplicate experiments, and error bars represent standard deviations.

**Fig. 4.**
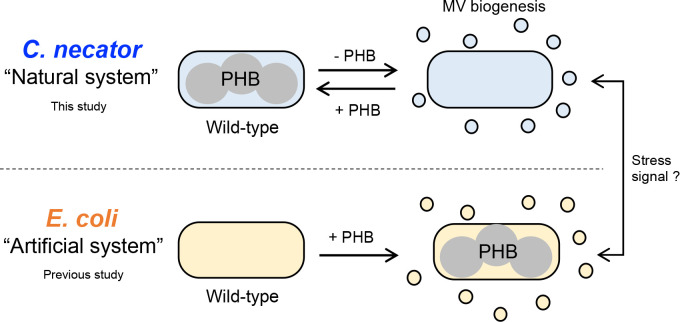
A schematic illustration showing contrasting patterns for PHB synthesis-associated MV biogenesis. Upper illustration (natural system): Once *Cupriavidus. necator* H16 (a PHB producer) did not produce PHB, MV biogenesis occurred. An inverse relationship between MV biogenesis and PHB production was demonstrated by a complementary experiment on the PHB synthesis gene. Lower illustration (artificial system): a contrasting pattern was observed for *E. coli* (PHB non-producer). MV biogenesis was triggered by installing PHB synthesis operon genes derived from *C. necator* H16 (termed PIA-MVP), as previously reported (Koh *et al.*, 2022). MV biogenesis may function as a stress signal for living microorganisms, and PHB synthesis may be in a normal state for *C. necator* H16, but not for *E. coli*.
